# Laser Shock Peening Improves the Corrosion Resistance of an E690 High-Strength Steel Cladding Layer

**DOI:** 10.3390/ma16165566

**Published:** 2023-08-10

**Authors:** Jiaxin Qin, Yupeng Cao, Weidong Shi, Zhengang Wang, Ming Qiu

**Affiliations:** 1 School of Mechanical Engineering, Nantong University, Nantong 226019, China; 2 Nantong COSCO Shipping Engineering Co., Ltd., Nantong 226006, China

**Keywords:** laser shock peening, electrochemical corrosion, E690 high-strength steel, microstructure

## Abstract

To investigate the effect of laser shock peening parameters on the corrosion resistance of an E690 high-strength steel cladding layer, NVE690 high-strength steel powder was selected for testing at various power densities of pulse lasers. The surface roughness and residual stress of the treated samples were measured, and the microstructure morphology of the sample surface was observed. The electrochemical corrosion tests were conducted with an electrochemical workstation to measure the electrometer polarization, obtain the impedance curve, and observe the electrochemical corrosion. As the laser power density increased, the surface grains of the E690 high-strength steel cladding layer continued to refine until nanocrystals formed, and the residual compressive stress on the surface increased. The residual compressive stress on the surface rendered the passivation film stable and dense; furthermore, the refinement of surface grains inhibited the initiation and propagation of microcracks. The positive shift of the corrosion potential increased from −1.004 to −0.771 V, the corrosion current density decreased from 114.5 to 5.41 μA/cm^2^, the radius of the impedance spectrum curve increased, and the peeling pits, as well as corrosion micropores on the surface, gradually became no longer evident after electrochemical corrosion. After laser shock treatment, the corrosion resistance of the cladding layer sample was substantially improved.

## 1. Introduction

E690 high-strength steel [1] is commonly used in pile leg materials and gears of marine platforms. The complex marine environment can lead to various failure forms of equipment [2,3], among which corrosion [4,5] is the most consequential. Laser shock peening [6,7] is a surface-strengthening process developed in the 1960s, which uses high-power laser irradiation to induce plasma shock waves to act on the target surface. Ning [8] investigated the effect of laser shock peening on the electrochemical corrosion performance of the IN718 high-temperature alloy. The results showed that, after laser shock peening, the corrosion resistance of the IN718 high-temperature alloy was improved, with a maximum improvement of about 84.07% compared to the untreated sample. Luo [9] studied the electrochemical corrosion behavior and pitting morphology of Mg-Al-Mn alloy coating after laser shock peening, and the results showed that the corrosion resistance of Mg-Al-Mn alloy coating was significantly improved after laser shock peening. Even at high concentrations of corrosive solutions, it can still prevent corrosion to a certain extent after laser shock peening. Wang [10] studied the effect of laser shock peening on the electrochemistry of AISI 420 Martensitic stainless steel in 3.5% NaCl solution. The results showed that carbide decomposition and microstructure nanocrystallization by laser shock peening contributed to the generation of Cr-rich passive film with better corrosion resistance. The surface current density was reduced by 98.1% and the pitting potential was increased by 89.5%. Numerous studies by scholars have shown that laser shock peening can cause plastic deformation of metal materials [11,12], proliferation of internal dislocations, precipitation and grain refinement [13,14,15], and increase the corrosion resistance of materials.

Metal corrosion tests can help protect sea industry platforms from failure [16]. Common corrosion test methods include exposure testing, mass loss testing, laboratory-accelerated testing, and electrochemical testing (which is especially useful) [17,18,19,20]. In this study, by using electrochemical methods, the corrosion resistance between the matrix, cladding repair layer, and cladding repair layer of E690 high-strength steel after laser shock peening was compared and studied. The impact of laser shock peening technical parameters on the improvement of corrosion resistance of E690 high-strength steel was evaluated, which can provide theoretical insights into improving the corrosion resistance of key components of offshore platforms.

This study aimed to simulate real working conditions and investigate the efficacy of repairing the corroded surface of E690 high-strength steel. The approach involved milling the corroded areas of E690 high-strength steel and subsequently using laser cladding technology with powder of similar composition to repair the damaged areas. And, various power densities of pulse lasers were used for laser shock peening of the cladding layer. The microhardness and residual stress of the treated samples were measured, and the microstructural morphology of the sample surface was observed. Electrochemical measurements were conducted on E690 high-strength steel, as well as the cladding layer before and after laser shock peening with an electrochemical workstation. Then, the surface morphology of the sample after electrochemical corrosion was observed by scanning electron microscopy (SEM), and the impact of laser shock peening on the corrosion resistance of the cladding layer of E690 high-strength steel was comprehensively analyzed.

## 2. Materials and Methods

### 2.1. Materials

The matrix material was E690 high-strength steel, and the cladding repair powder was NVE690 high-strength steel powder with a particle size of 45–105 μm. The purity was 99.9%. Table 1 shows the chemical composition of the matrix and powder. The dilution of substrate and claddings was approximately 12.76%.

### 2.2. Test Methods

**Figure 1:**
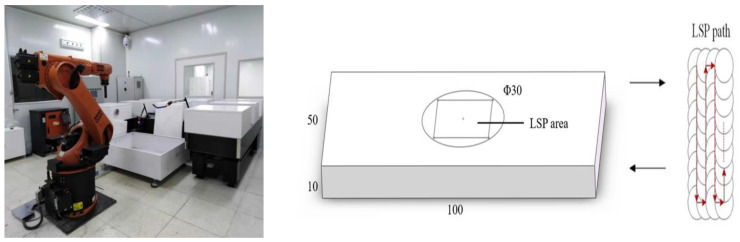
Laser Shock peening test and lapping scheme of laser shock region and spot.

The laser cladding experiment used a coaxial powder delivered by an AFS-C1280 experimental platform, using nitrogen as the protective gas and powder carrier gas. Parameters were as follows: protective airflow speed, 6 L/min; powder feeding pressure, 0.6 MPa; laser mode, TEM00; pulse width, 15 ns; laser power, 1000 W; spot diameter, 2 mm; overlap rate, 62.5%; powder feeding, 6 g/min; and laser scanning speed, 700 mm/min. After laser cladding, laser shock peening was undertaken in the 20 mm × 20 mm rectangular area of the sample center. The laser shock peening test used an Nd:YAG solid-state laser (SGR series, Beamtech Company, Beijing, China) with a wavelength of 1064 nm and a pulse width of 20 ns. Parameters were as follows: spot diameter was 2 mm; overlap rate, 70%; and energy, 3–7 J. The corresponding power densities were 4.77, 7.96, and 11.15 GW/cm^2^. The absorption layer used black tape and the constraint layer was deionized water. The sample surface was impacted three times. Figure 1 shows the laser shock peening test and the lapping scheme of the laser shock region and spot.

**Figure 2:**
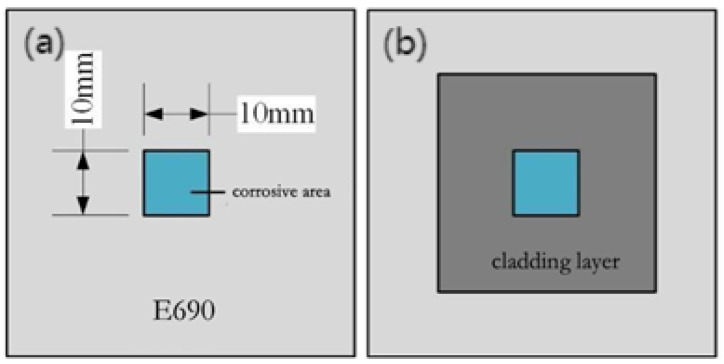
Schematic of the corrosion area of the sample: (**a**) E690, (**b**) cladding layer.

Regarding samples 1–5, power densities of 4.77-, 7.96-, and 11.15-GW/cm^2^ laser shock peening treatments were wire-cut to produce a 10 × 10 × 10 mm^3^ size specimen, respectively [Figure 2a,b, and Table 2]. Sandpaper was used to polish the surface of the sample; then, the sample was immersed in anhydrous ethanol for ultrasonic cleaning and subsequent air-drying.

### 2.3. Testing and Analysis

The residual stress on the surface of the sample after laser shock peening at various power densities was measured with an X-ray stress diffractometer Xstress3000 G2R. There were a total of five measurement points, with an interval of 2 mm between each point. The basic parameters for residual stress testing were as follows: collimation tube diameter, 1 mm; target material, Cr; Bragg angle, 156.4°; tube voltage, 30 kV; tube current, 6.7 mA; exposure time, 14 s; selection of Fe, ferrite (211) crystal plane type; and measurement by the lateral tilt method. The microhardness of the surface of the cladding layer before and after laser shock peening was tested using a digital micro Vickers hardness tester (TMVS-1 type, Beijing Shidai Zhifeng Technology Co., Ltd., Beijing, China). Five points were randomly selected on the surface of the sample, with a loading force of 200 g and a holding time of 15 s, to obtain the average microhardness value of the sample surface.

Field emission high-resolution transmission electron microscopy (Tecnai G2 F20, FEI Company, Hillsboro, USA) was used to observe the microstructure of the sample surface before and after laser shock peening under different laser energies. The TEM sample preparation process was as follows: First, the 5 × 5 × 2 mm^3^ sample was cut in the impact area of the sample by wire cutting, and the back of the sample was manually thinned to 0.5 mm with 400–2000# sandpaper. The thin slice sample with a diameter of 3 mm was removed from the surface of the sample by a circular nailing machine (Gatan 659), and the thin slice sample was fixed on a manual grinding disc (Gantan 653) with a fixed heating table (Gantan 653.40002) and hot melt adhesive, and the thin slice sample was ground along the back to 60 μm. The backside of the honed wafer was honed with a concave instrument (Gatan 656), and the honed wafer was thinned with a precision ion thinning instrument (Gatan 691).

The electrochemical corrosion tests were conducted with an electrochemical testing workstation (CHI660C, Shanghai Chenhua, Shanghai, China) by using a traditional three-electrode system. The electrochemical workstation used 3.5% aqueous NaCl, and the total exposed area of samples 1–5 was 1 cm^2^. The present authors cut out the 10 mm × 10 mm × 10 mm samples of the centers that were treated by laser shock peening. The laser shock peening surface is the surface that was corroded. Copper wire was glued to the back of the sample by using aluminum foil, and was placed in a soft film with a diameter of 35 mm. The other surfaces were sealed with a curing agent. During the electrochemical corrosion experiments, the relationship between the open-circuit voltage of the sample and the time (maximum of 300 s) was measured. Second, the polarization curve of the kinetic potential was measured. The initial potential was −1.5 V, the termination potential was 0 V, the scanning speed was 0.001 V/s, and the sampling interval was 0.01 s. Finally, the impedance spectrum curve was measured: initial potential, −1.5 V; high frequency, 100 kHz; low frequency, 0.1 Hz; and amplitude, 0.005 V.

The surface morphology of the samples after electrochemical corrosion was observed by scanning electron microscopy (Quanta 650F, FEI Corporation, Hillsboro, OR, USA) (SEM).

## 3. Test Results

### 3.1. Residual Stress

**Figure 3:**
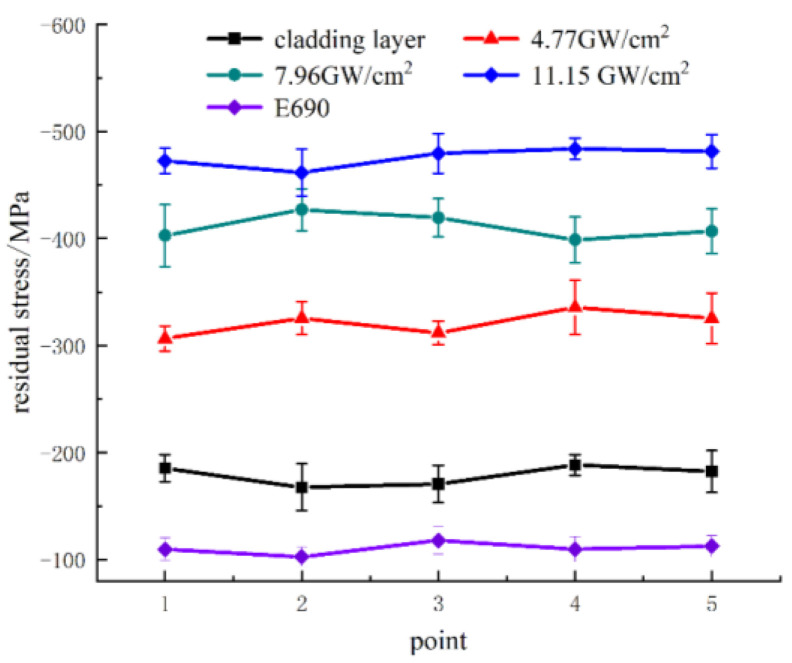
Surface residual stress of original specimens and samples after various laser shock peening treatments.

Figure 3 shows surface residual stress diagrams of the original sample and after various laser shocks. The surface residual stress of E690 of sample 1 was 110.4 ± 10.7 MPa. After laser cladding, the surface residual stress of sample 2 was −178.8 ± 16 MPa. When the power density was 4.77 GW/cm^2^, the average residual stress on the surface of sample 3 reached −321.0 ± 18 MPa; 80% higher compared with sample 2. At a power density of 7.96 GW/cm^2^, the average residual stress on the surface of sample 4 reached −410.9 ± 21 MPa; 28% higher compared with sample 3. At a power density of 11.15 GW/cm^2^, the average residual stress on the surface of sample 5 reached −475.8 ± 15 MPa; 16% higher compared with sample 4.

### 3.2. Microhardness

Table 3 presents the microhardness measurements obtained for E690 sample 1, cladding sample 2, and cladding samples 3–5 after laser shock peening. The initial cladding layer exhibited numerous defects, and the heat accumulation during the cladding process led to material softening, resulting in a lower microhardness (272.8 HV), compared to the E690 high-strength steel matrix (288.0 HV). The laser shock peening had no adverse effect on the microhardness index. In fact, the microhardness of the cladding layer significantly increased to 294.3 HV after laser shock peening with a power density of 4.77 GW/cm^2^, surpassing the level of the substrate. And, as the laser power density increased, the microhardness of the cladding layer continued to increase. The changes in surface microhardness and residual stress of the cladding layer were generally consistent, but residual compressive stress was more sensitive to laser shock peening.

### 3.3. Surface Microstructure

**Figure 4:**
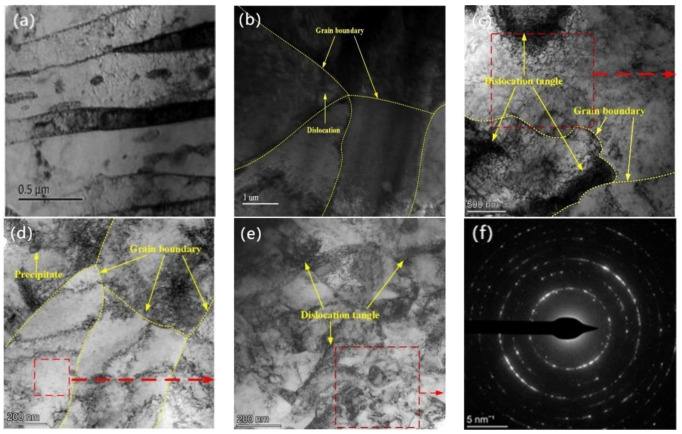
TEM morphological phase and electron diffraction pattern after laser shock at various power densities: (**a**) E690, TEM; (**b**) cladding layer, TEM; (**c**) 4.77-GW/cm^2^, TEM; (**d**) 7.96-GW/cm^2^, TEM; (**e**) 11.15-GW/cm^2^, TEM; (**f**) electron diffraction pattern.

Figure 4 shows the surface phase observed using transmission electron microscopy (TEM) after laser shock peening at various power densities. Figure 4a shows the TEM morphology of the surface of sample 1. The matrix of E690 high-strength steel was a pearlite composed of layered ferrite and cementite thin sheets. Figure 4b shows the TEM morphology of the surface of sample 2. The surface of sample 1 was generally an equiaxed crystal with a uniform internal structure, and the grain size was mainly distributed between 2 and 10 µm. Figure 4c shows the morphology as per the TEM of the sample 3 surface after laser shock peening at a power density of 4.77 GW/cm^2^. The grain size in the cladding layer was relatively large, and dislocation lines experienced difficulties in aggregating at the grain boundaries in a manner that formed blockages. Compared with Figure 4b, there was dislocation proliferation within the grains and high-density dislocation entanglement at the grain boundaries. Figure 4d shows the morphology as per the TEM of the sample 4 surface after laser shock peening at a power density of 7.96 GW/cm^2^. Dislocations proliferated extensively near the grain boundaries and within the grains, and the grain size decreased, mainly distributed from 0–2 µm. Compared with Figure 4c, the cladding layer grains were clearly refined. Figure 4e shows the morphology as per of the sample 5 surface after laser shock peening at a power density of 11.15 GW/cm^2^. Dislocations in the grains further proliferated, and dislocations generated by multiple dislocation sources intertwined in a manner that formed complex dislocation configurations. Complete large grains were continuously segmented through dislocations, forming numerous randomly oriented and uniformly distributed small grains < 100 nm. Figure 4f shows a selected-area electron diffraction analysis. There were concentric rings with various diameters, indicating that the cladding grains in this area formed randomly oriented and evenly distributed nanocrystals under the laser shock of 11.15 GW/cm^2^. Compared with Figure 4d, the surface grains of sample 5 were further refined. Thus, after laser shock peening at various power densities, the surface grains of the laser cladding layer were refined.

### 3.4. Cross-Section Microstructure

**Figure 5:**
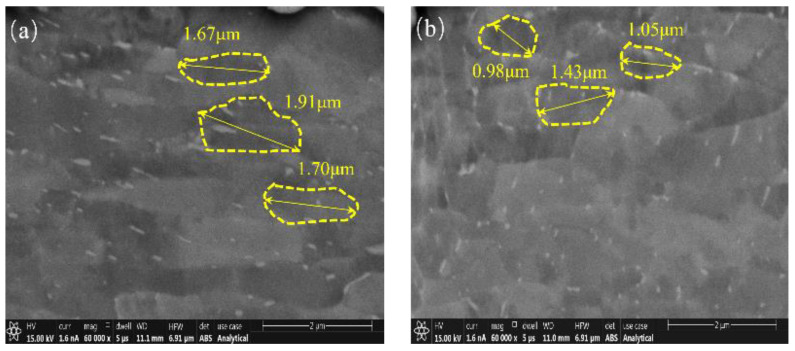
The microstructure of the cross-section of the cladding layer after laser shock peening. (**a**) 4.77-GW/cm^2^, (**b**) 11.15-GW/cm^2^.

The microstructure and morphology of the cladding layer are shown in Figure 4a. The overall structure of the cladding layer was equiaxed grains, and the average grain size of the surface layer was approximately 3.90 μm. Figure 5 shows the SEM morphology of the cross-section of the cladding layer after laser shock peening with different power densities. Using the intercept method to measure the grain size in Figure 5, it was found that the average grain size in Figure 5a was about 1.76 μm. Figure 5b shows an average grain size of approximately 1.15 μm. It indicated that, after laser shock peening, the surface layer of the cladding layer generated fine grain strengthening, forming a grain refinement layer of about ten micrometers. With the increase in laser shock peening power density, the grains were further refined, and the grain boundaries proliferate and connect into a network structure. The amount of energy required for corrosion to propagate along the grain boundaries increased, and corrosion crack propagation was suppressed.

### 3.5. Electrochemical Corrosion Performance

**Figure 6:**
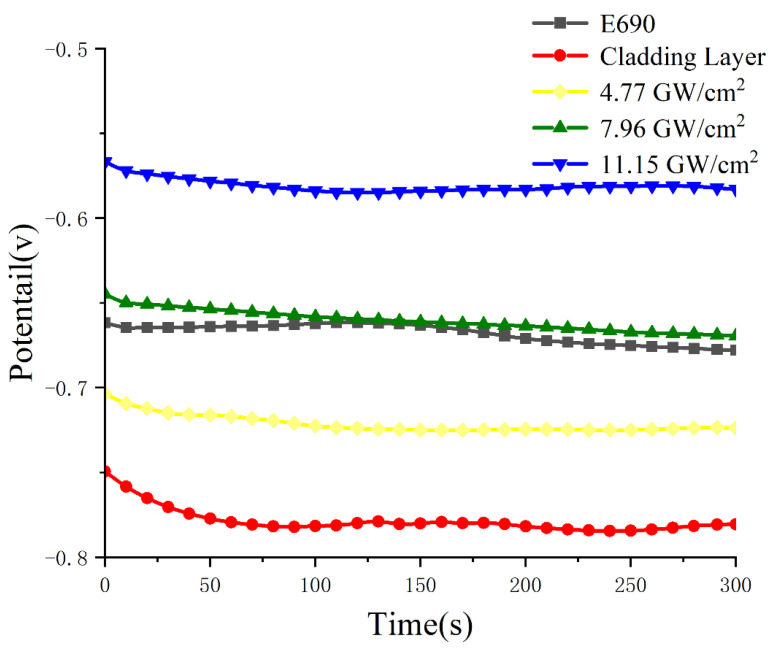
Open-circuit potential map before and after laser shock peening.

Figure 6 shows the relationship between the open-circuit potential and time of samples 1–5 in 3.5% aqueous NaCl. The variation in amplitude of the open-circuit potential of sample 1 of E690 high-strength steel was relatively small, with a slight fluctuation at 125 s, and finally gradually stabilized. Its open-circuit potential was −0.66 V vs. saturated calomel electrode (SCE). The open-circuit potential of sample 2 of the E690 high-strength steel cladding layer initially decreased rapidly, but gradually stabilized afterward. Its open-circuit potential was −0.75 V vs. SCE; a decrease of 12%, compared with sample 1, indicating substantial corrosion. The open-circuit potential values of samples 3–5 after laser shock peening increased, and the open-circuit potential curve was relatively stable. The open-circuit potential of sample 3 after laser shock peening at a power density of 4.77 GW/cm^2^ was −0.72 V vs. SCE; 4% higher than the open-circuit potential of sample 2. At 7.96 GW/cm^2^, the open-circuit potential of sample 4 was −0.64 V vs. SCE; 11% higher than the open-circuit potential of sample 3 and slightly higher than the open-circuit potential of sample 1. At 11.15 GW/cm^2^, the open-circuit potential of sample 5 was −0.56 V vs. SCE; a substantial increase of 12.5% compared with sample 4. In summary, the open-circuit potential vs. time curve gradually flattened after laser shock peening, and the anodic behavior became more stable. This is because of the improved microstructure of the laser shock treatment area, which reduced the corrosion of the cladding layer. In summary, the laser shock peening open-circuit potential vs. time curve gradually adopted a gentle slope, and the anode behavior became more stable. This is because of the microstructure of the improvement of the laser shock peening area, which reduced the corrosion of the melting layer.

**Figure 7:**
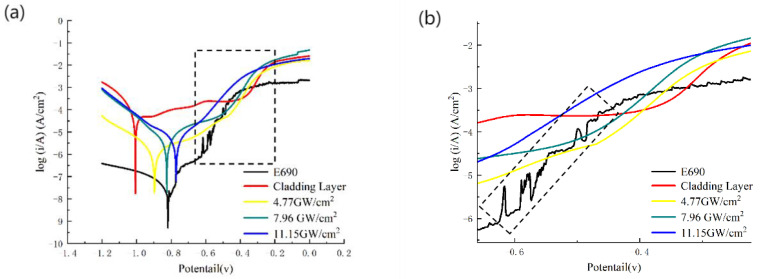
Dynamic potential polarization curve before and after laser shock peening: (**a**) polarization curve; (**b**) enlarged view of selected area.

Figure 7 shows potentiodynamic polarization curves of samples 1–5 in the corrosive solution, in which Figure 7b is an enlarged view of the box position in Figure 7a. Table 4 shows the relationship between the corrosion potential E_corr_ and corrosion current density J_corr_, obtained from the polarization curve in Figure 7. All of the curves are indicative of obvious passivation areas. The E690 substrate exhibited substantial current density fluctuations [Figure 7b]. After laser cladding and laser shock peening, there was no current density fluctuation, indicating that both laser cladding and laser shock peening afforded relatively stable corrosion. After laser shock peening at a power density of 4.77 GW/cm^2^, the corrosion potential of sample 3 was −0.897 V and the corrosion current density was 8.14 μA/cm^2^; a positive displacement of 0.107 V, compared with sample 2. The corrosion current density was much lower than that of the cladding layer without impact. At 7.96 GW/cm^2^, the corrosion potential of sample 4 was −0.825 V and the corrosion current density was 6.61 μA/cm^2^; compared with sample 3, the corrosion potential shifted forward by 0.072 V, which is equivalent to that of sample 1, and the corrosion current decreased by 1.53 μA/cm^2^. At 11.15 GW/cm^2^, the corrosion potential of sample 5 shifted in the positive direction to −0.771 V, the corrosion current density decreased to 5.41 μA/cm^2^, the corrosion potential further shifted forward by 0.054 V, and the corrosion current density decreased by 1.2 μA/cm^2^. In summary, after laser shock peening, the corrosion potential of the sample increased and the corrosion current density decreased, indicating weakened anodic dissolution performance and enhanced corrosion resistance. Compared with the other literature [21,22,23], the corrosion current density of the cladding layer decreased more significantly after laser shock peening in this experiment, with the corrosion current density increasing from 114.5 μA/cm^2^ and then dropping to 5.41 μA/cm^2^, with a significantly reduced corrosion rate and improved corrosion resistance.

**Figure 8:**
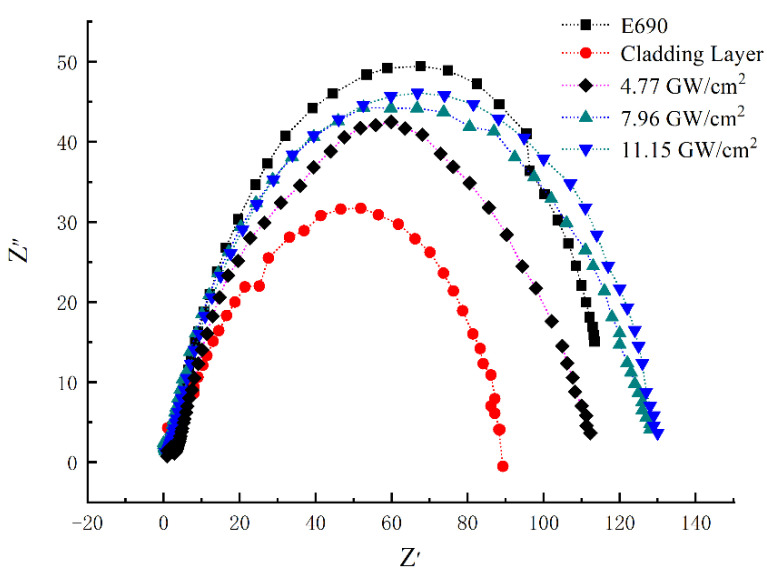
Nyquist plot of electrochemical impedance testing before and after laser shock peening.

Samples 1–5 were subjected to electrochemical impedance in 3.5% aqueous NaCl to obtain a Nyquist plot of electrochemical impedance (Figure 8). Z’ represents the real (resistive) component of the impedance whereas Z’’ represents the imaginary (capacitive) component of the impedance. The Nyquist curve of the impedance spectrum exhibits a semi-circular capacitive arc above the real axis, in which an increasing radius of the capacitive arc corresponds to a decreasing corrosion rate and increased corrosion resistance. The Nyquist curve radius of the impedance spectrum of sample 2 is relatively small, and the capacitive arc radius of Sample 1 is much larger than that of the cladding layer. The arc radius of sample 3 increased. The arc radius of sample 4 continued to increase and exceeds that of sample 1. The radius of the capacitive arc of sample 5 is slightly larger than that of sample 4. In conclusion, the impedance spectral radius of the sample increased after laser shock treatment, which indicates that, compared with no treatment, the samples had greater charge transfer ability and enhanced corrosion resistance after laser shock peening.

### 3.6. Surface Corrosion Morphology

**Figure 9:**
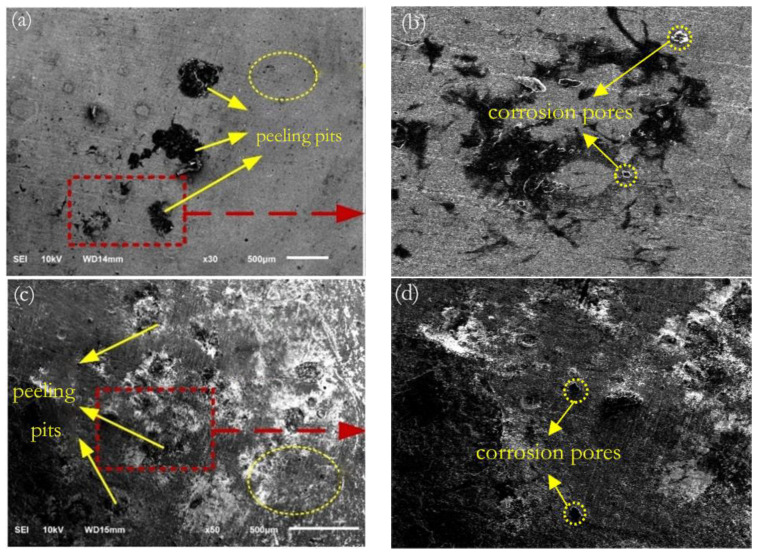

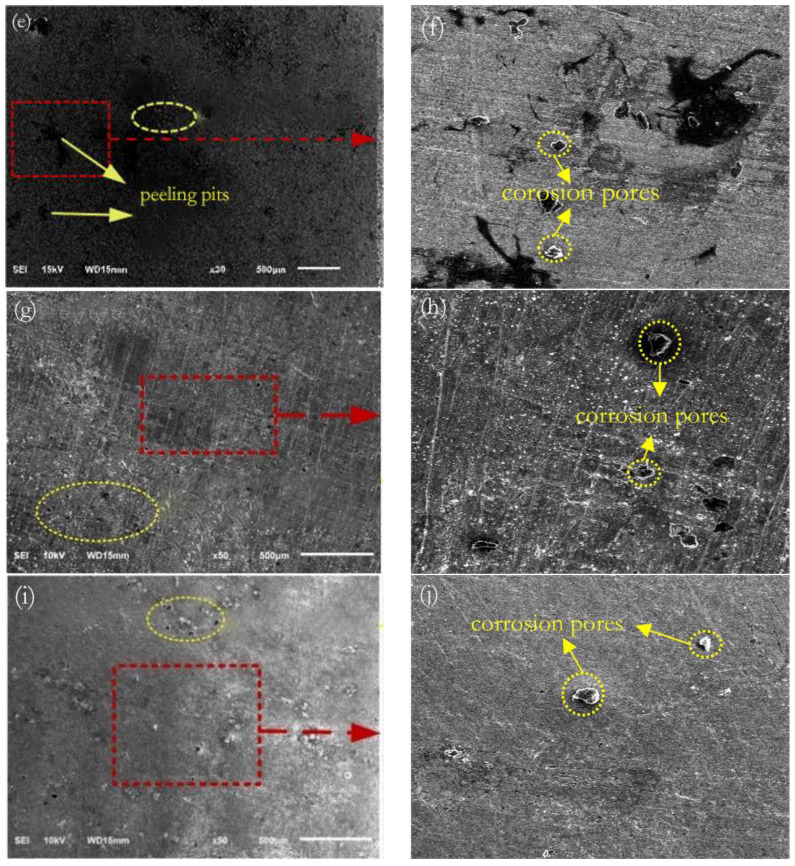
SEM morphology of corrosion before and after laser shock of the sample: (**a**) E690; (**b**) enlarged view (red box) of part a; (**c**) cladding layer; (**d**) enlarged view (red box) of part c; (**e**) 4.77 GW/cm^2^; (**f**) enlarged view (red box) of part e; (**g**) 7.96 GW/cm^2^; (**h**) enlarged view (red box) of part g; (**i**) 11.15 GW/cm^2^; (**j**) enlarged view (red box) of part i.

Figure 9 shows the post-corrosion morphology of samples 1–5 in 3.5% aqueous NaCl. The yellow area was corrosion holes. Figure 9a and Figure 8b show the morphology of E690 high-strength steel sample 1 after electrochemical corrosion as per SEM, in which Figure 9b is an enlarged view of the morphology of the red box area in Figure 9a. The surface was evenly distributed with many holes, yet there were some larger peeling pits.

Figure 9c and Figure 8d show the morphology of sample 2 of the E690 high-strength steel cladding layer after electrochemical corrosion as per SEM, in which Figure 9d shows an enlarged view of the morphology of the red box area in Figure 9c. The corrosion peeling pits on the surface of the cladding layer were relatively dense, with a small quantity of NaCl precipitate distributed near the peeling pits, and corrosion holes distributed near the peeling pits. Compared with sample 1, the corrosion pits in the cladding layer were widely distributed and the corrosion situation was substantial. Accordingly, the surface of the sample was composed of coarse grains, and there were residual compressive as well as tensile stresses on the surface, resulting in microcracks in the E690 high-strength steel cladding layer and weak corrosion resistance of the cladding layer.

Figure 9e and Figure 8f show the electrochemical corrosion morphology of the sample 3 surface after laser shock peening at a power density of 4.77 GW/cm^2^, in which Figure 9f is an enlarged image of the red box in Figure 9e. There were still a small number of peeling pits on the surface of sample 3, and many corrosion holes were distributed near the peeling pits. Compared with sample 2, the range of the peeling pits was reduced and the number of corrosion holes was reduced. Accordingly, the surface grains of the sample began to refine, the residual compressive stress increased, and the corrosion resistance was somewhat improved.

Figure 9g and Figure 8h show the electrochemical corrosion morphology of the sample 4 surface after laser shock peening at a power density of 7.96 GW/cm^2^, in which Figure 9h is an enlarged image of the red box in Figure 9g. Some corrosion pores of varying sizes were evenly distributed in the sample, whereas larger corrosion pores tended to develop toward spalling pits. Compared with sample 3, the peeling pits were largely no longer evident and the overall morphology was relatively complete. Accordingly, the surface grains of the sample continued to refine; furthermore, the residual compressive stress increased and was evenly distributed. This greatly improved the corrosion resistance of the cladding layer, surpassing the corrosion resistance of the E690 high-strength steel matrix.

Figure 9i and Figure 8j show the electrochemical corrosion morphology of the surface of sample 5 after laser shock peening at a power density of 11.15 GW/cm^2^, in which Figure 9j is an enlarged image of the red box in Figure 9i. Only a small number of corrosion holes were distributed in the cladding layer, and the surface of most areas was relatively intact. Compared with sample 4, the number of corrosion holes was substantially reduced and distributed in a certain area. Accordingly (and based on the literature [24]), as dislocations proliferated, the geometric dislocation interface divided the original coarse grains into small-angle sub-grain boundaries with various orientations. Continuous plastic deformation increased the grain orientation angle, and under the action of the geometric dislocation interface, large grains self-organized and differentiated into small grains, forming fine and uniform nanocrystals on the surface of the cladding layer. Furthermore, the distribution of residual compressive stress in each orientation was uniform, further enhancing the corrosion resistance of the surface of the cladding layer.

In summary, the surface corrosion of the cladding layer was relatively substantial, with a large number of corrosion peeling pits and corrosion holes, among which the corrosion resistance was the worst. After laser shock peening at a power density of 4.77 GW/cm^2^, the sample cladding layer gradually became uniform with grain refinement and residual compressive stress distribution, enhancing its corrosion resistance. The number of peeling pits and corrosion micropores on the surface of the cladding layer decreased. As the laser power density increased, the peeling pits on the surface of the sample cladding layer were no longer evident; furthermore, the number and size of the corrosion micropores decreased. After laser shock peening at a power density of 11.15 GW/cm^2^, nanocrystals were generated in the repaired layer of the sample, resulting in a uniform distribution of residual compressive stress. The corrosion resistance of the surface of the repaired layer of the sample was further improved.

## 4. Analysis and Discussion

Corrosion results from the oxidation of metals and reduction of environmental substances. Oxidation and reduction can be described by electrochemistry, which pertains to the charge-transfer response of the metal/environment interface, as well as the movement of electrically charged particles and electrons [25]. In the initial stage of the reaction, oxygen atoms dissolved in the electrolyte adsorb onto the surface of the cladding layer, forming a passivation film that isolates the contact between the cladding layer and electrolyte, slowing down the erosion of Cl^−^ ions. Over time, the passivation film on the surface layer ruptures and the steel is corroded, losing two electrons and becoming Fe^2+^, depositing Fe(OH)_2_ onto the surface of the cladding layer. The net ionic chemical reaction equations are as follows:

Anode Reaction Fe → Fe^2+^ + 2e^−^

Cathodic Reaction 2H^+^ + 2e^−^ → H_2_

Total Reaction Fe + 2H^+^ → Fe^2+^ + H_2_

**Figure 10:**
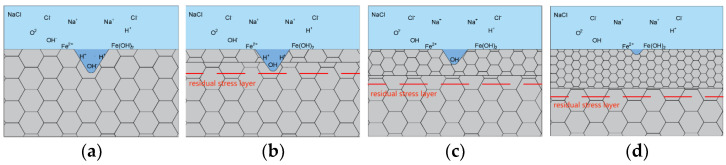
Corrosion resistance model of laser shock cladding layer on E690 high-strength steel: (**a**) cladding layer; (**b**) 4.77 GW/cm^2^; (**c**) 7.96 GW/cm^2^; (**d**) 11.15 GW/cm^2^.

To explain the impact of laser shock peening enhancement at various power densities on E690 high-strength steel melting layers in terms of the electrochemical corrosion performance, in accordance with the test results and laser shock peening enhancement theory, an electrochemical corrosion mechanism model (Figure 10) was established. Figure 10a shows the corrosion mechanism of the nonimpact cladding layer. The cladding layer is composed of coarse grains, with residual compressive and tensile stresses present on the surface. The corrosion current density is high, the corrosion is substantial, the surface morphology is substantially damaged, and there is a large number of peeling pits. Figure 10b shows the mechanism at a power density of 4.77 GW/cm^2^. The residual compressive stress on the surface of the material increases, the surface grains begin to refine, and the corrosion current density decreases. After corrosion, there are still peeling pits on the surface, but the size and number of peeling pits decrease, resulting in a decrease in the degree of corrosion. Figure 10c shows the mechanism at a power density of 7.96 GW/cm^2^. The surface grains of the cladding layer continue to refine. The residual compressive stress increases and is evenly distributed. After corrosion, the surface peeling pits are no longer evident and the corrosion resistance of the cladding layer is substantially improved. Figure 10d shows the mechanism at a power density of 11.15 GW/cm^2^. A large number of dislocations proliferate. Large grains self-organize and differentiate into small grains under the action of geometric dislocation interfaces. Fine and uniform nanocrystals form on the surface of the cladding layer. Residual compressive stress in each orientation is evenly distributed. After corrosion, there are only a small number of corrosion holes on the surface and the surface morphology is relatively complete. The corrosion resistance of the cladding layer is further strengthened.

In summary, residual compressive stress is generated in the cladding layer after laser impact, increasing the density and stability of the passivation layer formed during electrolysis, reducing the Cl^−^ ion-erosion rate. Residual compressive stress can suppress the generation and propagation of corrosion cracks, further alleviate the erosion damage of the electrolyte to the cladding layer, and reduce the corrosion rate. After laser shock peening, the refinement of surface grains leads to grain boundary proliferation. An increase in the grain boundaries corresponds to formation of a network structure, which increases the energy required for corrosion to propagate along the grain boundaries and suppresses the propagation of corrosion cracks.

## 5. Summary and Outlook

1. Laser shock peening was performed on the cladding layer using power densities of 4.77 GW/cm^2^, 7.96 GW/cm^2^, and 11.15 GW/cm^2^. As the laser power density increases, the surface grains of the E690 high-strength steel cladding layer continue to refine until nanocrystals are formed, and the surface residual compressive stress also increases. The surface residual stress increases from −178.8 ± 16 to −475.8 ± 15 MPa.
2. After laser shock peening, the fluctuation range of the open-circuit potential time curve of the cladding layer gradually decreases and tends to stabilize. The corrosion potential increases from −1.004 to −0.771 V, and the corrosion current density decreases from 114.5 to 5.41 μA/cm^2^. As the laser power density increases, the residual compressive stress on the surface renders the passivation film layer stable and dense, as well as less susceptible to Cl^−^ erosion, and the surface grain of the material is refined, inhibiting the initiation and propagation of microcracks, which delays surface corrosion. This is manifested by a positive shift in the corrosion potential, a decrease in the corrosion current density, an increase in the radius of the impedance spectrum curve, and a gradual elimination of surface peeling pits, as well as corrosion micropores. The corrosion resistance of the cladding layer sample substantially improves.
3. The present authors established a corrosion resistance model for laser shock peening of an E690 high-strength steel cladding layer, and elaborated on the relationship between laser shock peening technical parameters and the corrosion resistance. Regarding production of the passivation film, the microscale appearance, tissue composition, and laser shock peening parameters of a E690 high-strength steel melting layer passivation requires additional research. In the future, other process parameters such as spot diameter and impact frequency can be changed to observe the changes in the passivation film structure and cladding corrosion resistance after laser shock peening.

## Figures and Tables

**Table 1:** Chemical Composition of Matrix and Powder (Mass Fraction, %).

Element	C	Si	Mn	P	S	Cr	Ni	Mo	V	Fe
matrix	0.15	0.50	1.52	0.03	0.01	1.50	3.60	0.70	0.06	Bal.
powder	0.15	0.29	1.35	0.03	0.01	0.16	0.24	0.15	0.06	Bal.

**Table 2:** Sample corrosion number and power density.

Sample Number	Sample 1	Sample 2	Sample 3	Sample 4	Sample 5
Corrosive area	E690	cladding layer	cladding layer	cladding layer	cladding layer
Laser shock peening power density	untreated	untreated	4.77 GW/cm^2^	7.96 GW/cm^2^	11.15 GW/cm^2^

**Table 3:** Microhardness.

Samples	Microhardness/HV					Average Microhardness/HV
Sample1	286.7	292.6	281.9	284.1	294.6	288.0
Sample2	270.9	279.6	275.4	266.7	271.5	272.8
Sample3	297.8	288.9	296.5	294.7	293.6	294.3
Sample4	300.6	298.7	305.4	309.6	301.6	303.2
Sample5	308.9	312.6	311.6	312.4	309.8	311.1

**Table 4:** Corrosion parameters.

Sample	Ecorr/V	Jcorr/(μA∙cm^2^)	Epass/V	Ipass/(μA∙cm^2^)
E690	−0.810	0.089	−0.62 ± 0.15	0.84 ± 0.12
Cladding layer	−1.004	114.5	−0.83 ± 0.10	248.6 ± 82.2
4.77 GW/cm^2^	−0.897	8.14	−0.76 ± 0.12	24.82 ± 6.20
7.96 GW/cm^2^	−0.825	6.61	−0.71 ± 0.15	18.95 ± 3.36
11.15 GW/cm^2^	−0.771	5.41	−0.60 ± 0.16	15.58 ± 2.69

## Data Availability

Not applicable.
